# Self-Disclosure Here and Now: Combining Retrospective Perceived Assessment With Dynamic Behavioral Measures

**DOI:** 10.3389/fpsyg.2019.00558

**Published:** 2019-03-28

**Authors:** Hamutal Kreiner, Yossi Levi-Belz

**Affiliations:** ^1^ Department of Behavioral Sciences, Linguistic Cognition Lab, Ruppin Academic Center, Hadera, Israel; ^2^ The Lior Tsfaty Center for Suicide and Mental Pain Studies, Ruppin, Academic Center, Emek Hefer, Israel

**Keywords:** self-disclosure, objective measurement, behavioral measures, dynamic evaluation, linguistic analysis, vocal analysis, interpersonal interaction

## Abstract

Most previous research on self-disclosure (SD) focused on its perceived retrospective aspects using self-report questionnaires. Few studies investigated actual SD as reflected in interpersonal interaction. We propose a comprehensive approach that combines new objective and dynamic measures of SD that evaluate situated SD with the traditional measures that evaluate stable SD properties. As SD is essentially verbal, we build on linguistic parameters for assessing actual SD, including acoustic features such as intonation and fluency, and verbal features such as the particular choice of words. Critically, the new measures highlight SD *here and now* and may reveal transient situational factors that affect it, such as the dynamics of interpersonal interaction. Based on these measures, we propose a three-dimensional evaluation that can portray different profiles of SD and offer a better prediction of SD behavior in different situations. The theoretical and clinical implications of the proposed approach are discussed.

“Our ego is composed of the superimposition of our successive states.”

—Marcel Proust, Remembrance of Things Past, p. 622

## Introduction

Self-disclosure (SD), the communication of personal thoughts and feelings with another person) Jourard, 1971), has been conceptualized in the psychological literature in various ways and measured using various tools. Most of this research treated SD as a stable personality trait and accordingly focused on perceived retrospective measurements of self-disclosure. In this paper, we propose a novel approach that focuses on SD *in vivo* and examines potential tools for assessing the actual SD during ongoing interpersonal interaction. We posit that combining new objective and dynamic measures of SD with the traditional measures that evaluate stable SD properties will provide a more comprehensive understanding of SD.

SD is involved in many aspects of life such as the development of intimate relationships and coping with stress and traumatic events (for reviews see Kennedy-Moore and Watson, 2001; Frisina et al., 2004; Frattaroli, 2006). Decades of research show that SD contributes significantly to interpersonal relationships and may promote the development of liking, understanding, and intimacy (Carpenter and Freese, 1979; Berg and Derlega, 1987; Laurenceau et al., 1998). This is particularly true in virtual social networks where people get to know one another almost solely on the basis of SD (e.g., Joinson and Paine, 2007). Furthermore, SD is a beneficial behavior with positive impact on mental and physical health (Derlega et al., 1993). For example, it has been shown that even in an experimental setting where participants were asked to write about traumatic events, SD was associated with striking benefits to their physical health, including improved immune functioning (Pennebaker et al., 1988), reduction in health center visits (Pennebaker et al., 1990), and decreased self-reported upper respiratory problems (Greenberg et al., 1996). Other studies show that authentic SD to at least one significant other is a prerequisite for various aspects of psychological adjustment such as mental health, competence, self-efficacy, and social adaptation (Jourard, 1964; Greenberg and Stone, 1992) as well as to post-traumatic growth (Levi-Belz, 2015, 2016; Levi-Belz and Kreiner, 2016; Levi-Belz and Lev-Ari, 2019). By contrast, low levels of SD have been associated with a wide gamut of psychopathologies, including psychiatric illness, anxiety, low self-esteem, loneliness, hostility, and dissatisfaction with life (Jourard, 1971; Harvey and Omarzu, 1997; Wei et al., 2005; Kahn and Garrison, 2009), and even suicidal behavior (Levi et al., 2008; Horesh et al., 2012; Levi-Belz et al., 2014).

The historic roots of the SD concept can be found “at the heart of psychotherapy” (Stiles, 1995, p. 71), where clients’ revealing of their personal thoughts, emotions, and conflicts is an essential component of the therapeutic process (Farber, 2006). Based on the interpersonal psychoanalytic theory of Harry Stack Sullivan (1953), Sidney Jourard conceptualized the tendency to reveal personal information as SD (Jourard, 1958; Altman and Taylor, 1973). Following Jourard, several scholars emphasized various aspects of SD. For example, Altman and Taylor (1973) highlighted the social function of SD, describing it as a process in which people let themselves be known by others. Derlega and Grzelak (1979) focused on the content rather than on the social function of SD, defining it as “any information exchange that refers to the self, including personal state, dispositions, events in the past, and plans to the future” (p. 152). Other researchers stressed the reflective aspects of SD, describing it as descriptive, evaluative, and affective information about the self (Morton, 1978). Following Altman and Taylor (1973), Omarzu (2000) emphasized the multidimensional nature of SD and defined three main dimensions of SD behavior: the breadth of SD, as reflected in the number of topics disclosed; the depth of SD, as reflected in the level of intimacy of the disclosure; and the duration of SD, as reflected by the sheer amount of time devoted to disclosure.

While different scholars emphasize different aspects of SD, they seem to agree on two important features. The first is the verbal nature of SD (Cozby, 1973; Omarzu, 2000), and the second is that SD is a behavior that occurs within an interpersonal interaction. Despite this consensus, most of the tools developed to evaluate SD have not been designed to appraise the behavioral aspects of SD as reflected *in vivo* in actual interpersonal interaction. In what follows, we briefly review some of the tools used to appraise SD, and propose new tools designed to evaluate the actual SD. The new tools we propose address the verbal nature of SD in order to derive verbal and acoustic parameters from the actual interpersonal interaction in which SD occurs.

## The Assessment of Self-Disclosure

Various tools have been developed to assess SD. The early approach to SD assumed that, like other personality traits, it is a relatively stable faculty. Accordingly, the tools developed to evaluate it required participants to rate themselves with regard to their general tendencies (e.g., Jourard, 1971). Later tools, however, focused on more situational descriptions (e.g., Chelune, 1976). In general, it seems that the different tools are indicative of changes in the conceptualization of SD and the objective of its measurement. In this section, we briefly discuss the existing tools for assessing SD, and then discuss new potential tools based on methodology from the fields of computational linguistics and acoustic speech analysis.

### Self-Report Assessment Tools and Their Limitations

Perhaps the first attempt to offer an operational way to appraise SD was made by Jourard and Lasakow (1958), who developed a self-report scale named the Jourard Self-Disclosure Questionnaire (JSDQ; Jourard, 1971). In the JSDQ, participants are asked to rate to what extent they speak with other people about everyday topics that include attitudes and opinions, interests, study and work, personality, finance, and body. Each topic is sampled by several items, and participants are asked to rate their tendency to disclose information about each item on a 5-point Likert scale, ranging from “not at all” to “in full and complete detail.” Participants may be asked to rate their tendency to disclose personal information to different people such as father, mother, same-sex friend, opposite-sex friend, and lover. This self-report questionnaire allows researchers to evaluate SD on an array of topics and with a variety of people from different social circles. Another frequently used questionnaire is the Distress Disclosure Index (DDI; Kahn and Hessling, 2001) that focuses on the disclosure of negative emotions. The DDI measures the tendency to disclose or conceal personally distressing information, thoughts, personal problems, and unpleasant emotions across time and situations. Other similar tools are the Self-Concealment Scale (Larson and Chastain, 1990) and the Self-Disclosure Index (Miller et al., 1983).

A slightly different tool for assessing SD is Chelune’s (1976) Self-Disclosure Situations Survey (SDSS) which focuses on situations. The SDSS presents participants with 20 social situations designed to sample their willingness to disclose personal information in a variety of social interactions with varying levels of intimacy. The 20 items represent four groups of situations with different target persons. Each group is comprised of five items sampling different settings scaled on intimacy. Thus, instead of rating general tendencies, participants rate their tendency to disclose information in a particular situation to a particular partner. While this tool employs situational descriptions to evaluate SD, these are nonetheless generic situations and thus the evaluation is not an actual situation-based assessment of SD.

While the traditional tools vary on several aspects, such as topics, breadth of disclosure, or the target partner, they all share two important features: the basic conception that SD is a stable personality trait, and the ensuing reliance on self-report questionnaires. Self-report SD questionnaires have important practical advantages: they are easy and quick to administer, they are highly reliable, and their validity as a subjective measure is very good. Moreover, the use of self-report SD questionnaires has proven to be very productive in studies that employed it as a dependent variable or even manipulated it as a pseudo-causative independent variable in many fields of psychology. Thus, accumulating research has demonstrated that such questionnaires are highly informative and sensitive to individual differences in SD (e.g., Horesh et al., 2012; Kahn et al., 2012). In sum, self-report tools aim to appraise the persistent tendency, and, by definition, their context sensitivity is low, as they were not designed to measure transient factors involved in SD. Hence, self-report tools reflect a generalized, cumulative, and retrospective perception of SD, but they lack sensitivity to situational factors that may affect it.

Critically, however, it has been shown that situational variables can affect participants’ level of SD. For example, environmental factors such as desk size and room spaciousness can facilitate or impede SD (e.g., Chaikin et al., 1976; Okken et al., 2013). Specific characteristics of the recipient are also important, for example, disclosure is expressed more easily toward women and between people of comparable age and status (e.g., Cappella, 1981). Moreover, a meta-analysis of studies investigating the relationship between liking and SD showed a dynamic relationship whereby people disclose more to those whom they initially like, and they like others more as a result of having disclosed to them (Collins and Miller, 1994). In addition, it has been shown that positive affect is associated with higher levels of SD (Ignatius and Kokkonen, 2007; Forgas, 2011). Thus, it seems that different factors that affect the discloser’s state of mind influence the likelihood of sharing information. In view of these and similar findings Antaki et al. (2005) criticize the a-contextual approach in measuring SD and discuss SD “as a situated interactional practice” (p. 181).

A different line of criticism relates to the discrepancy between actual behavior and participants’ self-report of that behavior. For example, in a study that investigated communication skills comparing self-report measures, trained observers’ ratings, and behavioral measures, Carrell and Willmington (1998) found a complex relationship between the three types of measures that did not reveal significant correlations between self-report measures of communication apprehension and actual communication competence as measured behaviorally. One explanation for the lack of correlation is that, unlike the behavioral measures, self-report measures may be biased by participants’ generalized self-perception.

Thus, it seems that a-contextual tools such as self-report questionnaires cannot be assumed to capture the complex and dynamic nature of SD. In order to obtain a more comprehensive understanding of SD, additional aspects need to be evaluated, in particular behavioral and situational aspects, including those related to the interpersonal interaction within which SD occurs.

### Assessment Tools for Situated Self-Disclosure

Recent years have brought a gradual change in the perception and measurement of self-disclosure. Increasing attention has been paid to the situated aspects of SD, and several attempts have been made to develop tools with higher context sensitivity for measuring the “actual self-disclosure” as exhibited within interpersonal interaction (e.g., Zhao et al., 2012).

**Figure 1:**
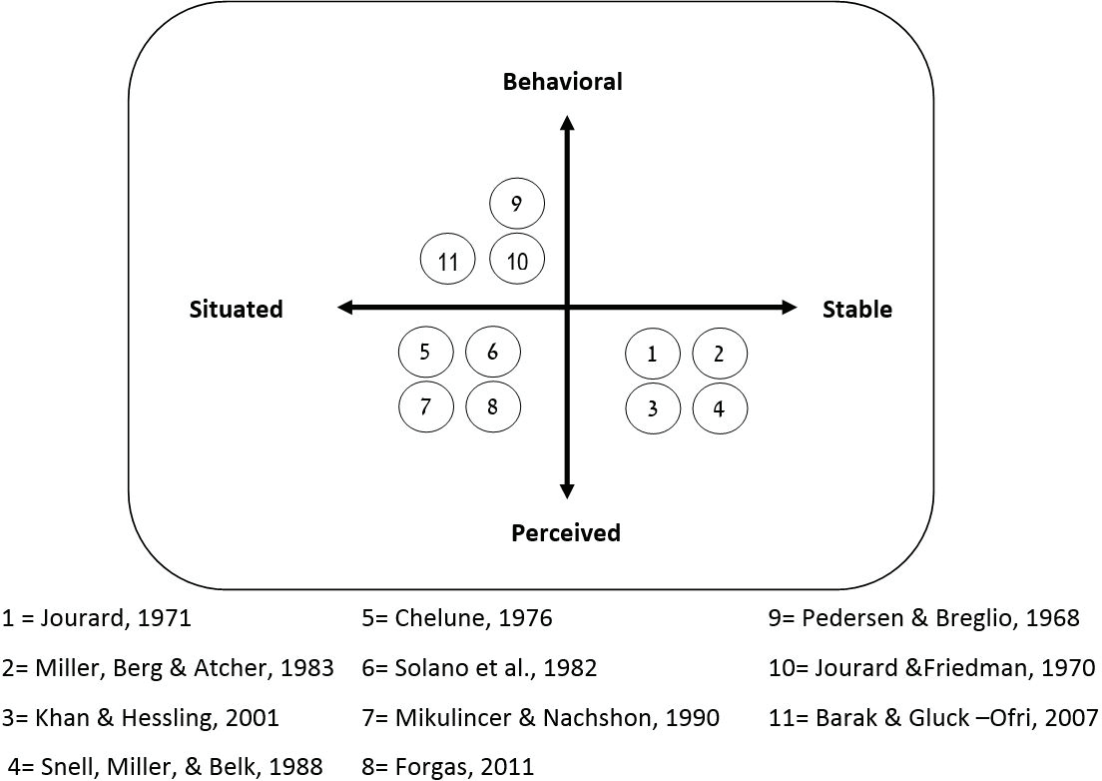
Mapping of SD assessment tools onto a two-dimensional array representing the stable vs. situated conceptual dimension and the behavioral vs. perceived operational dimension.

Close examination of the different assessment tools used in recent SD studies reveals two important dimensions on which they vary. The first dimension is associated with the conceptualization of SD—to what extent can SD be considered a relatively stable personality trait and to what extent ought it be conceived of as a situated process. The second dimension relates to the measurement of SD—to what extent do the assessment tools objectively measure the actual behavior of SD, and to what extent do they measure perceived SD using subjective ratings (either participant’s self-perception, as reflected in self-report questionnaires, or the perception of an observer, such as a therapist or independent “blind” judges, rating the subject’s SD). Figure 1 maps different assessment tools onto a two-dimensional array representing the behavioral-perceived and the situated-stable dimensions. It is important to note that this is not a systematic review of all methods but rather a selective set of examples aimed to show the different dimensions of SD evaluation.

The classic studies of SD did not make these distinctions. Self-report SD questionnaires were considered as a convenient operational interpretation of the theoretical conception of SD as a stable personality trait. Jourard (1971) assumed that stable personality traits underlie the individual differences in self-disclosure behavior. Nevertheless, Jourard’s questionnaire (JSDQ; Jourard, 1971) evaluated SD independently to different partners (e.g., close friend, parent), assuming that SD may vary depending on the quality of the interpersonal interaction. Thus, SD may be described more adequately as a behavior modulated by both stable personality traits and situated processes. SD self-report questionnaires were designed to appraise the generalized and lasting SD tendency, but were insensitive to fluctuations in SD; consequently, they were not suitable for assessing situational factors that modulate SD, such as the dynamics of the interpersonal interaction. Studies that focused on SD in actual interactions between people applied two different approaches in their attempts to measure situated SD, one relying on ratings reported by independent judges and the other relying on behavioral measures.

In studies employing rating measures to evaluate *actual* SD, independent judges are typically presented with verbal statements produced by participants during interpersonal interaction, and asked to rate their content. For example, Solano et al. (1982) asked participants to rate how well they now knew another person with whom they had conducted a short conversation, and used these ratings as a measure of the *actual* SD. In another study (Mikulincer and Nachshon, 1991), participants conducted a short conversation with a stranger, and the recorded statements were presented to independent judges who rated their “descriptive intimacy” (the extent to which intimate facts were revealed) and their “evaluative intimacy” (the extent to which emotions or judgments were expressed). Forgas (2011) used a similar method in a study about the effect of mood on self-disclosure. Two independent judges were presented with five self-disclosing statements produced by participants and were asked to rate them on four disclosure characteristics: intimacy, variety, abstractness, and valence (see also Kahn and Garrison, 2009). Similar methods based on content ratings were also used in studies about self-disclosure in online forum messages on the Internet (e.g., Barak and Gluck-Ofri, 2007).

This new method proved advantageous in two ways. First, aiming to evaluate actual SD in interaction stimulated the discussion concerning the conceptual dimensions of SD and their behavioral expressions; these were required in order to determine what aspects of the behavior should be evaluated (e.g., verbal communication, body and facial gestures, eye-contact) and what rating scales should be applied (e.g., intimacy, valence). Second, judges’ ratings that focused on situated SD provided a tool for exploring the effects of situational factors, like mood (Forgas, 2011) or medium of communication (Barak and Gluck-Ofri, 2007), on SD. Moreover, judges, unlike participants in the interactive situation, were not as subjectively biased as subjects assessing themselves. Nevertheless, this method has some drawbacks. Even independent judges are prone to bias, both by their own history and personality and by the content of the material they judge (Tausczik and Pennebaker, 2010). Not incidentally, even with trained judges it is hard to obtain high levels of agreement (e.g., Barak and Gluck-Ofri, 2007, reported agreement ranging from 0.71 to 0.77). Second, although this methodology was developed to evaluate situated SD, it is still based on perceived impression and thereby provides only an off-line evaluation that cannot record moment-to-moment fluctuations in SD. Yet, even one of Jourard’s earliest studies indicated that factors that may fluctuate on a moment-to-moment basis such as eye-contact, minimal physical contact, and personal distance play an important role in the interpersonal dynamics and affect SD (Jourard and Friedman, 1970).

A different approach to evaluating situated SD focuses on behavioral measures. Pedersen and Breglio (1968) were perhaps the first to apply this approach to investigate SD. They asked participants to answer questions about different topics (similar to the JSDQ topics) and simply counted the number of words participants produced in response to each question as an indicator of SD. Similarly, Jourard and Friedman (1970) measured the duration of time spent in self-disclosure. More recently, this approach was used in studies of computer-mediated communication to investigate reciprocity in SD (e.g., Joinson, 2001; Barak and Gluck-Ofri, 2007). Thus, behavioral measures such as duration and word count of verbal disclosure were regarded as measures of the quantitative aspect of SD. One of the major advantages of such behavioral methods is their capacity to record subtle and short-term fluctuations in behavior. Hence, they would appear to be very useful for investigating situational factors that modulate SD.

To conclude, the brief overview presented here demonstrates that there are many potential ways to assess SD, and that different tools reflect different aspects of SD. Self-report questionnaires that reflect long-lasting perceived impressions (mapped onto the lower-right corner of Figure 1) focus on the stable aspects of SD and are compatible with the initial conceptualization of SD as a personality trait. Measures of situated SD designed to evaluate SD in context focus on the actual SD and are more compatible with the view of SD as a dynamic process that should be evaluated in the context of interpersonal interaction. These include both perceived measures (mapped onto the lower-left corner of Figure 1) and behavioral measures (mapped onto the upper-left corner of Figure 1). Whereas perceived measures can be informative about contextual factors that affect SD, behavioral measures offer a more objective evaluation, recording moment-to-moment fluctuations that reflect the dynamic aspects of SD. This promising approach seems to offer an important perspective on the moment-to-moment factors, operating in the interpersonal interaction, that modulates SD, such as eye-contact, personal distance, and reciprocity. Yet the tool box required to explore this direction is currently limited, consisting of only two behavioral measures, namely word count and discourse duration. The development of psycholinguistic research methods and computational linguistics tools leads more and more scholars to posit that objective analysis of verbal behavior can be a valid measure of personality and personal states (e.g., Groom and Pennebaker, 2002; Pennebaker et al., 2003). In what follows, we outline some ideas for developing new tools based on such methods that will allow objective, temporally detailed, and multidimensional evaluation of situated SD.

### Analyses of Verbal Communication as Assessment Tools for Situated Self-Disclosure

Almost any behavior can be interpreted as disclosing the self. However, language is often described as the “most common and reliable way for people to translate their internal thoughts and emotions into a form that others can understand” (Tausczik and Pennebaker, 2010, p. 24). It is only natural then to assume that verbal expression plays a central role in the actual act of self-disclosure, and indeed most of the definitions of SD refer to verbal communication as the medium of SD (e.g., Jourard, 1971; Cozby, 1973; Chelune, 1979). In view of its key role in SD, we propose that verbal communication may be analyzed in different ways to provide rich and reliable information that can be used for evaluating situated SD. We focus on two different aspects of the verbal communication: first, we discuss potential parameters of verbal communication, and second, the acoustic expression of the spoken message.

#### Verbal Expression

Psychological evaluation has always used *qualitative* analysis of verbal expressions produced in evaluation settings such as interviews and projective tests. In the last two decades, however, the development of computerized algorithms for Natural Language Processing (NLP) offers novel *quantitative* methods for analyzing verbal expressions. These methods have proven useful for psychological evaluation (for a review see Tausczik and Pennebaker, 2010) and have yielded informative findings in a variety of contexts, such as the evaluation of distress and depression (e.g., Rude et al., 2004), social status (Gonzales et al., 2010), responses to upsetting (Boals and Klein, 2005), or even traumatic events (D’Andrea et al., 2012).

##### Pragmatic Analysis

Antaki et al. (2005) argue that the voluntary intention to disclose information and the degree to which it can be considered significant personal information are critical characteristics of SD (see also Adler and Towne, 1999) that can only be determined by analyzing its context. They use discourse analysis tools to demonstrate how pragmatic parameters such as shared information and intention contribute to the evaluation of SD in an utterance. For example they present a statement such as “I’m a relief teacher” that can be considered as voluntary disclosure of significant personal information about the speaker’s job in a certain context. However, if delivered in the context of schedule arrangement (e.g., “…but you know I’m a relief teacher, I’ve been asked to teach on Thursday…”) this statement is not disclosing any new or personal information. Hence, they suggest that pragmatic parameters such as intention, shared information, and point of view are essential for evaluating SD in discourse (Antaki et al., 2005). Although, Antaki et al.’s (2005) study used qualitative analysis, quantitative discourse analysis of such parameters can be conducted using computational linguistics algorithms.

##### Word Count

Tausczik and Pennebaker (2010); p. 25 posit that “The words we use in daily life reflect who we are and the social relationships we are in.” Hence, they proposed that systematic quantitative analysis of the particular words used in a message may yield important psychological information for both diagnostic and therapeutic purposes. Indeed, a longitudinal study that used such analysis on texts written following the September 11 attack in 2001 revealed linguistic content characteristics that predicted lasting Traumatic Stress Disorder (PTSD) symptoms, (D’Andrea et al., 2012).

Most SD studies employing quantitative computational methods focused on general word count. However, different types of words have been associated with different social and psychological aspects of the verbal interaction. The broadest distinction is among content words (e.g., nouns, regular verbs, adjectives, and adverbs) and function words (e.g., prepositions, pronouns, articles, conjunctions, and auxiliary verbs). According to Tausczik and Pennebaker (2010), content words reflect what people are saying, whereas function words reflect how they say it. Thus, while the former may be informative about the disclosed themes and emotions, the latter may convey important information about the cognitive and social processes underlying the style of the interaction.

##### Total Number of Words

The total number of words produced by a person in an interaction (or in one turn in the interaction) seems to reflect the volume of SD, such that higher total word counts have been associated with higher SD (e.g., Pedersen and Breglio, 1968; Joinson, 2001; Barak and Gluck-Ofri, 2007). This measure has been shown to be very informative regarding various aspects of SD in interpersonal interaction. For example, reciprocity is considered as an important factor in SD, such that the recipients of disclosure tend to respond by disclosing comparable levels of information about themselves (e.g., Berg and Derlega, 1987). This claim was supported by a study that used total word count and showed that among female reactors in an online forum, the counts of original messages were highly correlated with the counts of the reaction messages (Barak and Gluck-Ofri, 2007). Thus, response-based word counts provided a very good tool to evaluate *in vivo* the dynamic nature of SD within an interpersonal interaction.

However, using total word counts as a measure of SD may have two caveats. First, Pedersen and Breglio (1968) found that females did not use more words to describe themselves than males, yet they disclosed more intimate information. This finding suggests that the total number of words reflects only one aspect of SD while neglecting other aspects, such as the intimacy or valence of the disclosed information. Another problem is that additional characteristics of social interaction such as dominance in the conversation have also been associated with word count (Tausczik and Pennebaker, 2010). On one hand, these findings highlight the weakness of total word count, suggesting it cannot be assumed to reflect SD per se. Conversely, the word count measure may be viewed beneficially because it reveals the relationship between SD and other socio-psychological factors involved in interpersonal interaction.

##### Pronouns

The relative counts of different pronouns are indicative of social-cognitive processes, such as social-attention, social status, and trustworthiness (Tausczik and Pennebaker, 2010). For example, in a study that examined prisoners, who were instructed to either lie or tell the truth about videos they had watched, a lower rate of third-person pronouns was a significant predictor of deception (Bond and Lee, 2005). In addition, pronoun counts in texts such as political speeches and medical interviews were reliably associated with depression level (Weintraub, 1981, 1989). Later studies have further shown that people who experience physical and emotional pain use more first-person singular pronouns. This observation was interpreted as suggesting that frequent use of these pronouns is indicative of self-oriented attention (e.g., Rude et al., 2004). Indeed, Barak and Gluck-Ofri (2007) used counts of first-person pronouns (e.g., I, me) as an index of the amount of text that participants produce to describe themselves (see also Derlega and Berg, 1987; Joinson, 2001). These are few examples of the accumulating evidence suggesting that pronoun count and pronoun ratios (e.g., the relative proportion of first- vs. third-person pronouns) may be highly informative regarding the interpersonal processes underlying SD interaction.

##### Emotion Words

Counts of emotion words are indicative about the emotional expression in SD, and the relative counts of positive (e.g., love, nice, sweet) and negative (e.g., ugly, dirty, hurt) emotion words reflect the valence of the emotions disclosed. A study that examined emotional expression in describing life-events showed that indeed more positive words were used in describing positive compared to negative events and the reverse was true when participants described negative events (Kahn et al., 2007). Moreover, in a study that examined the verbatim of women who described incidents of domestic violence (Holmes et al., 2007), increased use of both positive and negative emotion words was associated with reports of increased physical pain during the writing sessions. This finding suggests that a higher rate of emotion words is indicative of a higher degree of immersion in the traumatic event, which is reflected in increased perception of physical pain. These and other studies join to suggest that the analysis of emotion words may be informative of changes in the emotional aspects of SD. Potentially, different parameters derived from the ratio of emotion words, their valence (positive, negative), and their value (weak, mild, strong) may be respectively indicative of the volume, valence, and value of the emotions that participants experience and choose to share during SD.

In summary, accumulating evidence strongly support the idea that computational algorithms that draw on verbal communication can reveal the links between verbal parameters and psychological evaluation (e.g., LIWC; for a review see Tausczik and Pennebaker, 2010). To apply this methodology to SD, further research is required to explore the association among these verbal parameters and the relevant aspects of SD as assessed by other tools. However, other more complex parameters should also be considered. For example, analyses that draw on sentence features such as syntactic complexity (e.g., Snowdon et al., 1996) or discourse features such as focus, and new vs. shared information (e.g., Antaki et al., 2005) may also prove informative.

#### Vocal Expression

The development of advanced methods for acoustic analyses in the last decade allows researchers to explore acoustic aspects of speech and associate them with psycho-physiological processes underlying verbal interaction. Initial attempts to use the vocal aspects of spoken messages to examine psychological processes were based on listeners’ judgments (e.g., Wiseman and Rice, 1989). Physical analysis of the vocal qualities of spoken communication is a relatively novel method for measuring participants’ experience in an interpersonal interaction *in vivo*. Although, to the best of our knowledge, such measures have not been used to investigate SD, they have been extensively studied in research investigating other psychological processes, and in particular emotions (e.g., Scherer et al., 2003). Here we focus on three vocal parameters, namely loudness, intonation, and speech rate that can be objectively measured by analyzing the corresponding acoustic features.

##### Loudness

The acoustic feature subjectively perceived as speech loudness refers to the intensity level of the voice, represented as the *amplitude* of the acoustic signal (measured in dB). Intuitively, we feel that when people are reluctant to disclose information, they tend to speak quietly or even whisper, but this intuitive observation has yet to be systematically and objectively examined. Studies that focused on emotions showed that vocal expressions of high-arousal emotions such as anger and fear were associated with high-intensity voice whereas sad and bored vocal expressions were associated with low-intensity voice (Scherer et al., 2003). More generally, a study that examined a large variety of emotions found that variation in emotion was highly correlated with the variance in speech amplitude (Pfitzinger and Kaernbach, 2008). Most relevant to SD, accumulating evidence suggests that speaking about personally meaningful events may evoke higher levels of sympathetic activation and potentially lead to increased amplitude range (e.g., Rochman et al., 2008). Thus, acoustic analysis of amplitude variation may reflect emotional aspects of SD.

##### Intonation

The acoustic feature subjectively perceived as the speaker’s intonation draws mainly on the *fundamental frequency (F0)* of the speaker’s voice (e.g., Rosen and Rosen, 1992). Several studies have established the relationship between the acoustic features of intonation and perceptual judgments of emotional activation, emotional valence, and emotional dominance (e.g., Rochman et al., 2008; Amir et al., 2010). For example, in Rochman et al.’s (2008) study, participants expressed anger and sadness, either induced by an experimental manipulation or produced naturally in an emotion-focused analogue-therapy session. The findings revealed differential acoustic signatures for anger and sadness, in which anger was associated with larger intonation variability compared to neutral baseline speech or to sadness. Interestingly, the findings further revealed that increased variations in imperceptible F0-perturbation were associated with sadness. This finding suggests that acoustic analysis may provide us with implicit indicators of emotion that cannot be consciously perceived, in addition to the vocal features that can be explicitly perceived.

##### Speech Rate

The acoustic feature subjectively perceived as speech rate may be calculated as either the number of *words per minute* (WPM) or by measuring syllable or phone rate (e.g., Amir et al., 2010). These measures are susceptible to changes in the activation of the sympathetic nervous system (e.g., Titze, 2000), hence previous studies used them mainly as indicators of emotion (Scherer and Banse, 1991). For example, increased WPM values were exhibited by speakers that moved from sad or neutral emotion to anger (Rochman et al., 2008). Moreover, speech rate was correlated to the activation, valence, and dominance of the emotions expressed by spacers as evaluated judges (Amir et al., 2010). Indirect indication that speech rate may be associated with SD comes from a study revealing that in comparison to normal speech, during seductive interaction between male and female partners, speech rate was increased. The authors argued that the seductive interaction involves increased SD aimed to establish intimacy. Hence although SD was not directly measured, they suggest that changes in speech rate are associated with changes in SD (Anolli and Ciceri, 2002).

In summary, we propose that acoustic analyses of the vocal expression of the spoken message may provide additional parameters for the evaluation of SD which are not necessarily captured by the verbal parameters. These parameters seem to be highly informative of emotions, interpersonal interaction, and other psychological aspects of communication that characterize the actual SD. Moreover, these parameters have two important advantages. First, some of the measures have been claimed to be more universal than the computational linguistics parameters described above because the latter are language-specific and thus susceptible to local and social variations in language use (Scherer et al., 2001). Second, at least some acoustic aspects of speech are controlled by the sympathetic nervous system; consequently, they may reflect automatic uncontrolled processes not moderated by social and cognitive biases. Further research is required to study these and additional measures (e.g., patterns of silence, disfluencies) and explore their potential contribution to the assessment of SD (but see Levi-Belz and Kreiner, 2016).

## Toward an Integrated Self-Disclosure Assessment

The brief review of verbal and acoustic measures presented above suggests that alone, none of these measures can capture the complex nature of SD, as each measure reflects only certain aspects of it. For example, imagine you meet a colleague in the cafeteria, and you ask: “how are you.” If she or he is not inclined to discuss their feeling, they might answer fluently and briefly “I am fine, thank-you very much for asking, and how are you today?”. By contrast, if they are more willing to disclose their thoughts or feelings they might start slowly with “I am fine,” then pause, then say, “but I didn’t sleep well,”, then take their time to consider how much more they would like to disclose, and after another pause continue, “I’m concerned about the revision of a paper” (Levi-Belz and Kreiner, 2016). This example demonstrated that general word count is indicative of the volume of SD communication but tells us nothing about how emotional, personal, or reflective it is. By contrast, intonation and rhythm may be indicative of the emotional intensity of SD communication but not of its content. This seeming drawback may develop into an advantage if these measures are pooled into a combined assessment tool that will offer a multidimensional perspective on SD.

Currently, some of the measures described above need to be further explored and their relationship to different aspects of SD need to be further established. Nevertheless, in a recent study (Levi-Belz and Kreiner, 2016), we have made an initial attempt to evaluate SD in interpersonal interaction using combined linguistic tools. Importantly, to evaluate the validity of such tools, we examined to what extent SD as reflected in linguistic measures was correlated with subjective (self-reported) SD and with SD as judged by others. In this study, participants were interviewed, and the recorded interviews were submitted to verbal (classified word counts) and vocal (speech fluency measures) analyses, and presented to judges who rated the speakers’ SD. Linguistic parameters provided good prediction of the situated judged SD, whereas self-reported SD measures failed to predict the judges’ ratings. These preliminary findings suggest that linguistic SD measures may provide a valid evaluation of situated SD, and call for further research to examine the scope of their validity and extend it, and to explore the gap between the long-term self-reported SD and the situated SD as reflected in linguistic SD evaluations (Levi-Belz and Kreiner, 2016).

Thus, in this last section, we outline our proposal for a comprehensive evaluation of SD that integrates both traditional self-report SD scales such as the JSDQ (Jourard, 1971) and a combined measurement of situated SD evaluation based on verbal and acoustic measures as described above. We hope that future research inspired by this proposal will develop this approach and empirically test our proposal.

### Combined Measurement of Situated SD

The development of a combined tool for evaluating situated SD may pursue either a data-driven approach or theoretically driven one. A data-driven approach may be agnostic to theoretical assumptions and rather begin with the rich array of data provided by the different acoustic and verbal measures. It can use statistical tools such as Multi-Dimensional Scaling (MDS) to explore the shared and unique variability of the different measures, outline the major dimensions of SD, and then examine to what extent they fit the SD theoretical literature.

By contrast, a theoretically driven approach should begin with a definition of the major dimensions of SD and use statistical tools such as confirmatory factor analysis (CFA) to map the different SD measures onto these dimensions. The current review suggests that verbal and acoustic measures may capture three important dimensions of SD, namely, *WHAT, HOW,* and *HOW MUCH*. This mapping may be tentatively associated with Omarzu’s (2000) *WIDTH, DEPTH,* and *DURATION* dimensions, with the WHAT referring to *WIDTH*, the HOW to *DEPTH*, and the HOW MUCH to an extended version of Omarzu’s (2000) *DURATION* dimension. We believe, however, that any attempt to map the different measures onto dimensions of SD should be guided by empirical evidence.

**Figure 2:**
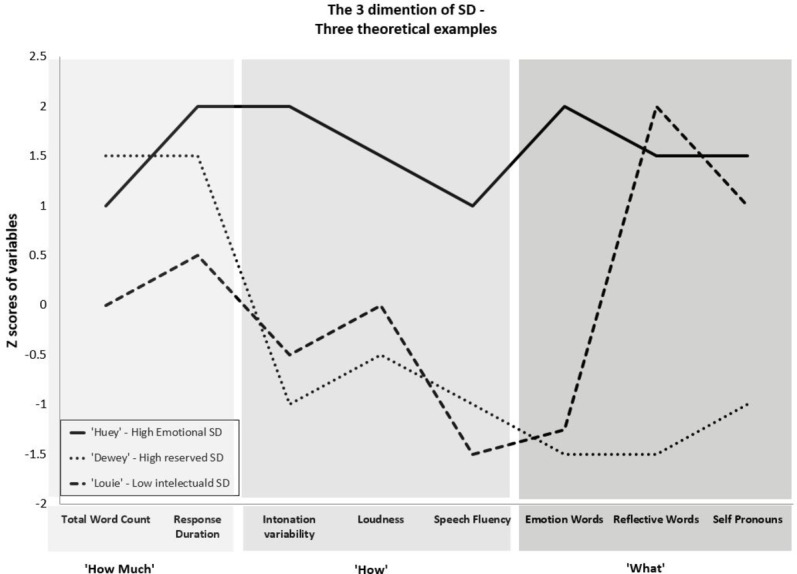
Three-dimensional SD profiles: Three hypothetical examples of different SD styles.

Based on this tentative mapping, it is possible to build a three-dimensional profile of SD. The *HOW MUCH* dimension refers to the volume of information disclosed and, as demonstrated in previous studies, can be measured by total word count and the duration of the spoken communication. The *WHAT* dimension refers to the content conveyed in the SD communication, namely, what topics are discussed, what emotions are disclosed, and what characters are referred to (e.g., the self, significant others). It may be evaluated by content measures such as counts of emotion words, reflective verbs (e.g., expect, understand), and pronouns (e.g., I, me, you, we). Finally, the *HOW* dimension refers to the way this content is conveyed, and may be reflected in the acoustic measures. For example, measures such as intonation and loudness may be indicative of the intensity of experienced and expressed emotions, while speech fluency (as measured for example by number and duration of silent pauses) may be informative regarding the ease or difficulty in disclosing information. The raw values from all these measures can be standardized and presented on a combined profile as demonstrated in Figure 2. The combined evaluation then provides a complex profile of a person’s situated SD rather than a single value on a high-to-low continuum.

Figure 2 presents hypothetical SD profiles generated by the different measures of situated SD for three different individuals—“Huey,” “Dewey,” and “Louie.” Both Huey and Dewey show high values on the volume measures such that if we only used volume measures they would have been classified as reflecting high levels of disclosure. However, when examining the complete profile, we can see very different SD styles. Huey exhibits high levels of intonation variability and loudness while Dewey exhibits low levels on these measures. Moreover, Huey shows high counts in all lexical classes, and Dewey shows low word counts on emotion words and self-pronouns with relatively high counts on reflective verbs. Thus, while Huey and Dewey’s profiles both reflect a lot of talking, Huey seems to be able to disclose his distress and express his emotions whereas Dewey’s verbosity seems to cover reserved speech with less emotional and personal content. Thus Huey’s SD style may be described as highly emotional and intimate, whereas Dewey’s SD style may be described as pseudo-disclosure, possibly avoiding intimacy and suppressing distressing emotions and experiences. Finally, Louie exhibits medium values on the total word counts, response duration, intonation, and loudness measures with very low values on fluency, and emotion words, but high counts of reflective verbs. His profile suggests a more pensive and intellectual SD style, he seems to be willing to share his experience but has difficulties in articulating his emotions.

These hypothetical profiles demonstrate how the three-dimensional evaluation of situated SD may highlight the complex nature of SD. These profiles may represent different SD styles exhibited by three different individuals. However, they may also represent different SD styles exhibited by one individual at different time points. For example, a patient starting a therapeutic interaction somewhat reluctant to talk, gradually talking more but with a reserved non-emotional intellectual SD style, that may turn at a certain point into an intimate emotional SD. Thus these profiles can reveal changes in SD patterns that can be associated with situational factors such as the dynamic aspects of interpersonal interaction.

The proposed tools have three important advantages. First, evaluation can be performed during natural interaction and does not require a formal setting of evaluation nor does it require self-report questionnaires that may generate a “test” setting. Second, it provides an objective evaluation (as it is computerized) less susceptible to biases of diagnosticians, or to intentional and motivational biases of the individual tested than self-reported SD or to potential. Finally, this tool is highly sensitive to real-time changes during interaction so it can shed light on particular characteristics of the interaction that affect the individual SD *in situ*. While the advantages are very clear, it is important to note that the development of such tool requires computationally intensive algorithms. Moreover, although psychologists intuitively interpret patients’ verbal and vocal expressions, the algorithms underlying the proposed tool are opaque and not intuitive to psychologists, hence they may doubt its face validity and therefore be reluctant to use it. These disadvantages may hinder the development and implementation of such evaluation tools; in order to facilitate this process, it is important to integrate these novel methodological tools with more familiar and widely used tools.

### Integrating Perceived Self-Report SD and Situated SD Evaluations

Situated SD profiles as described above reflect actual SD during interpersonal interaction, but as such they are not designed to capture the long-lasting tendency to disclose personal information. Previous studies that used SD measures were interested either in the actual SD or in the stable SD, tendency only few studies examined the correlation between actual SD, as measured by word counts, and self-reported SD, as measured by JSDQ (Pedersen and Breglio, 1968; Levi-Belz and Kreiner, 2016). These studies indicate that actual SD and self-reports of the stable SD are not highly correlated and suggest that they should be considered as complementary measures of SD rather than alternate or clashing measures. Using both measures may have important implications both at the theoretical and at the practical—clinical levels.

At the theoretical level, future research that will explore individual differences in the gaps between the actual and self-perceived SD may shed light on the psychological processes that generate it. Such dissociation between behavior and self-perception may be indicative of problematic personality development, for example, or inflexible use of defense mechanisms. Understanding the psychological processes that generate the gap can promote our understanding of the contribution of SD to coping with life challenges and conflicts.

Moreover, exploring the relationship between patients’ long-lasting tendency to disclose and their actual SD during therapeutic interactions may have important clinical implications. Recent research (e.g., Aderka et al., 2012; Bohn et al., 2013) suggests that in therapeutic interactions, a positive change is often not revealed in gradual and systematic progress from one meeting to the next, rather, a sudden gain is revealed at a certain point in the therapeutic process. As the actual SD style is often a key indicator of such change, the ability to evaluate it may be critical for understanding the underlying processes that triggered the change. Critically, patients with different levels of SD tendency may respond differently to different therapeutic approaches. For example, a patient with low SD tendency may show a substantial increase in the actual SD following some SD from the therapist, whereas a patient with high SD tendency may not need this, or even show reduced actual SD in response to the therapist’s SD. In addition, the therapist may learn from the discrepancy between the actual and self-perceived SD about the vulnerability of the patient in interpersonal interactions and their difficulties in developing intimate relationships.

## Conclusion

Self-disclosure is a complex behavior modulated by stable personality traits as well as by transient situational factors. To obtain a comprehensive appraisal of SD, we must be able to measure the actual situated SD as well as the stable SD tendency. Measuring actual SD presents a number of methodological challenges. Clearly, self-report methods are inadequate, as they interfere with the natural interaction. In this paper, we proposed the development of new tools that may enable better measurement of the transient, situated aspects of SD. Our proposal followed from the widely accepted view that SD involves sharing of personal and emotional information about the self through verbal communication (Jourard, 1971; Cozby, 1973; Omarzu, 2000; Chaudoir and Fisher, 2010). Consistent with this view, the measures proposed here are derived from the verbal communication of SD and aim to evaluate to what extent this communication is personal, emotional, and concerns the self.

Several of the measures reviewed in this paper have been employed in other contexts to evaluate situated SD as well as other psychological processes. It seems then that such measures may reflect general non-specific psychological processes. We posit, however, that it is possible to combine the different measures into an integrated assessment tool specifically tuned to SD. We believe that by exploring the shared and the unique contributions of various acoustic and verbal measures to the assessment of SD, future research would promote a multidimensional appraisal of SD. Such appraisal could be envisaged to complement the measures of stable perceived SD tendency, reveal its interaction with situational factors such as the dynamics of the interpersonal interaction, and allow us to predict actual SD behavior in given situations. We believe that the development of computerized tools to measure the acoustic and verbal parameters and implement an integrated moment-to-moment evaluation in real-time is just a matter of time. These tools would be helpful in different contexts in which *in situ* objective evaluation is needed. For example, on help-lines where the only information available about the caller is the recorded conversation, such tools can be used to evaluate their tendency to SD during the conversation, and enhance risk assessment (e.g., for suicidal ideation or attempts). Applying such tools in crisis interventions may promote therapists’ understanding of participants’ immediate responses to the dynamics of the intervention, helping them to adjust it individually as they progress, in order to obtain better results. Finally, these tools may be helpful for malingering assessment in which self-reported emotions and distress are suspected to be exaggerated, or even false, attempting to obtain undeserved gains.

“The words we use in daily life reflect what we are paying attention to, what we are thinking about, what we are trying to avoid, how we are feeling, and how we are organizing and analyzing our worlds” (Tausczik and Pennebaker, 2010; p. 25). By drawing on verbal and acoustic measures derived from the verbal SD communication, it should be possible to develop new and exciting tools which are sensitive to temporal fluctuations in SD during actual interpersonal interaction. This may have important implications both for the research and theory of SD as well as for clinical contexts. Researchers would be able to evaluate the effects of therapists’ responses on patients’ SD in clinical settings such as help-line or therapeutic sessions. Such research may provide evidence-based insights with important implications for the theoretical debates about interpersonal relationship in counseling and psycho-therapy (Gunlicks-Stoessel and Weissman, 2010).

## Author Contributions

HK and YL-B wrote the first draft of the manuscript, conducted the review of the literature, and were responsible for data extraction and quality assessment. HK and YL-B contributed to the interpretation of data, critically revised the manuscript, and approved the final version.

### Conflict of Interest Statement

The authors declare that the research was conducted in the absence of any commercial or financial relationships that could be construed as a potential conflict of interest.
